# Hybrid Reinforcement of Carboxymethyl Cellulose Films with Nanocellulose, Bentonite, and Ceramic Industry Waste: Structural, Thermal, and Morphological Characterization

**DOI:** 10.3390/polym18202485

**Published:** 2026-10-12

**Authors:** Mustafa Zor, Sümeyra Köse, Erdal Kara

**Affiliations:** 1 Department of Nanotechnology Engineering, Zonguldak Bülent Ecevit University, Zonguldak 67100, Türkiyeerdalkara@beun.edu.tr (E.K.); 2 Çanakcılar Seramik San. ve Tic. A.Ş., Creavit R&D Center, Gökçebey, Zonguldak 67670, Türkiye; 3 Department of Handicrafts, Gökçebey M. M. Çanakçı Vocational School of Higher Education, Zonguldak Bülent Ecevit University, Gökçebey, Zonguldak 67670, Türkiye

**Keywords:** carboxymethyl cellulose, nanocellulose, bentonite, ceramic waste valorization, hybrid biopolymer films

## Abstract

In this study, biodegradable hybrid polymeric films were developed by reinforcing a carboxymethyl cellulose (CMC) matrix with nanocellulose (NC), bentonite, and ground fired ceramic waste recovered from the vitrified ceramic industry. Seven formulations were prepared by the solvent casting method, and their structural, thermal, thermomechanical, and morphological properties were characterized by Fourier transform infrared (FT-IR) spectroscopy, differential scanning calorimetry (DSC), thermogravimetric analysis (TGA/DTGA), dynamic mechanical analysis (DMA), and scanning electron microscopy with energy-dispersive X-ray spectroscopy (SEM-EDX). DSC analysis revealed endothermic peaks at 240–248 °C corresponding to the onset of thermal degradation, while DTGA curves showed a two-stage degradation behavior for all formulations. The incorporation of inorganic reinforcements markedly improved thermal stability: the temperature at 10% mass loss (T_10_%) increased from 150 °C for pure CMC to 207 °C for the ceramic-waste-containing films, and the highest T_50_% value (397 °C) and char yield (40.2%) were recorded for F7 (80% CMC, 10% NC, 10% ceramic waste), confirming a thermal barrier effect. DMA results showed that F4 (85% CMC, 10% NC, 5% bentonite) exhibited the highest storage and loss modulus values, whereas excessive bentonite loading reduced stiffness through agglomeration. FT-IR spectra indicated that interactions between the components were governed by hydrogen bonding and physical mixing rather than covalent bonding, and SEM-EDX analyses revealed more homogeneous filler dispersion at low loading levels. Accordingly, F4 and F7 were identified as the optimum formulations for dynamic mechanical stiffness and thermal stability, respectively.

## 1. Introduction

Polymers are high-molecular-weight macromolecules formed by joining many monomers through covalent bonds [1,2]. Their controllable structure allows properties such as strength, flexibility, and processability to be tailored, making them essential across packaging, automotive, construction, textiles, electronics, and biomedical industries [3,4,5]. Since the mid-20th century, petroleum-based synthetic polymers such as PE, PP, PVC, and PS have dominated due to their low cost and strength [3], but their slow degradation causes serious environmental problems through microplastic formation [6,7,8]. According to the literature, only about 9% of the 8.3 billion tons of plastic produced between 1950 and 2015 has been recycled [7], and UNEP [9] warns that continued production trends will intensify ecological pressure and threaten sustainability goals [4,5].

To address these issues, research on biopolymers—renewable, biodegradable materials with lower carbon footprints—has accelerated [5,10,11]. Natural polysaccharides and proteins such as starch, cellulose, chitosan, alginate, pectin, and gelatin are widely studied for sustainable material development [10,11,12]. Among these, cellulose is the most abundant renewable polysaccharide, valued for its strength, biocompatibility, and biodegradability, though its poor water solubility limits processability [13,14,15]. Carboxymethyl cellulose (CMC), obtained by etherifying alkali cellulose with sodium monochloroacetate, converts this insoluble structure into a functional, water-soluble biopolymer [12,14]. Its degree of substitution (DS) governs solubility, viscosity, and film-forming behavior [16], and its biodegradability, viscosity-building capacity, and oxygen-barrier properties make it valuable in packaging, drug delivery, wound dressings, and tissue engineering [17,18].

Polymeric films, commonly produced via solvent casting due to its simplicity and low cost [19,20], are widely applied in food packaging, coatings, and biomedical materials, with CMC standing out for its film-forming ability [12]. However, pure CMC films suffer from low mechanical strength, high moisture sensitivity, and weak water-vapor barrier properties, motivating research into composite films reinforced with nanocellulose, clay minerals, and metal oxide nanoparticles [17,18].

Nanocellulose (NC)—including cellulose nanocrystals, nanofibrils, and bacterial nanocellulose—offers high surface area, low density, and superior mechanical properties, forming strong hydrogen bonds with polymer chains that enhance mechanical strength, thermal stability, and barrier performance [15,21,22,23]. Excessive NC loading, however, can cause agglomeration and reduce homogeneity, requiring optimized dosing [23].

Bentonite, a montmorillonite-rich layered clay with high cation exchange capacity and swelling ability [24], similarly improves mechanical strength, thermal stability, and gas-barrier properties when incorporated into CMC matrices, owing to strong interfacial hydrogen bonding and intercalated/exfoliated structures that extend diffusion pathways [25,26,27,28].

Ceramic industry waste, rich in SiO_2_ and Al_2_O_3_, is increasingly recovered as a filler in cement and polymer composites due to its hardness, chemical stability, and contribution to circular-economy goals [29,30,31,32,33]. Its use in CMC-based biopolymer films, however, remains largely unexplored.

Although considerable attention has been paid to CMC-based composite films reinforced with nanocellulose or clay minerals, the literature has predominantly focused on individual reinforcement systems rather than comparative hybrid strategies involving different inorganic fillers. Nanocellulose has been extensively investigated as a reinforcement phase for cellulose-based polymers due to its high aspect ratio, large specific surface area, and ability to form hydrogen bond networks with polysaccharide matrices. Similarly, bentonite and other layered silicate clays have been reported to improve the mechanical, thermal, and barrier properties of CMC-based systems through the formation of strong interface interactions and meandering diffusion pathways [25,26]. In contrast, while ceramic waste has been investigated primarily as a recycled mineral component in cemented materials, its application as a functional reinforcement in biodegradable CMC-based films has remained relatively limited [34].

Building on this rationale, the present study combines nanocellulose, bentonite, and ground ceramic powder within a CMC matrix to develop a hybrid biopolymer film. The aim is to produce non-toxic, water-soluble, biocompatible films with enhanced mechanical, thermal, and morphological properties, offering a sustainable alternative to petroleum-based plastics, and to characterize the resulting films’ structural, thermal, and morphological behavior.

## 2. Materials and Methods

### 2.1. Materials

The carboxymethyl cellulose sodium salt (CMC, low viscosity) used in this study was supplied by Sigma-Aldrich, Molsheim, France. The nanocellulose (NC) used in this study was prepared following the procedure described by Candan et al. [35], using bleached softwood kraft pulp as the starting material. The resulting nanocellulose consisted of string-like particles roughly 20 nm in diameter and about 1 µm in length. It was produced from bleached wood pulp via the TEMPO-mediated oxidation method, in which the oxidation mediator 2,2,6,6-tetramethyl-1-piperidin-1-oxyl converts some of the alcohol groups along the cellulose chain into carboxylic acid groups. The negative charges introduced by these carboxyl groups generate ionic repulsion between the fibrils, which promotes their separation. The bentonite was supplied by Esan Eczacıbaşı Endüstriyel Hammaddeler San. ve Tic. A.Ş., İstanbul, Türkiye. The ground ceramic waste was supplied by Çanakcılar Seramik Sanayi ve Ticaret A.Ş., Zonguldak, Türkiye.

### 2.2. Preparation of Hybrid Polymeric Films

The formulation values given in Table 1 were used to prepare the hybrid polymeric films. For each film, a beaker of appropriate volume was selected, and the solid materials were weighed into the beaker in the specified amounts. Then, 100 mL of distilled water was added. The mixture was heated to 90 °C on a hot plate while being continuously stirred with a magnetic stirrer bar. Stirring and heating were continued until the solid components were completely dissolved and a homogeneous appearance was obtained. After dissolution was complete, the mixtures were poured into Petri dishes. The dishes were dried in an oven preheated to 40 °C for 4 days. The resulting films were removed from the dishes and cut to the desired dimensions with scissors for the characterization studies.

### 2.3. Characterization of Hybrid Polymeric Films

The degradation temperatures (Td) of the films were determined by differential scanning calorimetry (DSC) using a Perkin Elmer DSC 8000 instrument, Perkin Elmer, Waltham, MA, USA; the analyses were carried out from 39 °C to 415 °C at a heating rate of 10 °C/min under a nitrogen atmosphere. The thermal stability and mass losses of the films with increasing temperature were investigated by thermogravimetric analysis (TGA) and differential thermogravimetric analysis (DTGA) using a Perkin Elmer Pyris 1 instrument, Perkin Elmer, Waltham, MA, USA from 30 °C to 750 °C at a heating rate of 10 °C/min under a nitrogen atmosphere. The thermomechanical properties of the films were examined by dynamic mechanical analysis (DMA) using a Perkin Elmer DMA 8000 instrument, Perkin Elmer, Waltham, MA, USA, from 25 °C to 300 °C at a heating rate of 5 °C/min, applying a sinusoidal deformation amplitude at a frequency of 1 Hz with a displacement of 0.1 mm. The chemical structures of the films were determined by Fourier transform infrared (FT-IR) spectroscopy using a Perkin Elmer Frontier instrument, Perkin Elmer, Waltham, MA, USA, with spectra recorded in the wavenumber range of 500–4000 cm^−1^. The cross-sectional morphology (SEM) and particle distribution (EDX) of the films were examined using a FEI Quanta FEG 450 scanning electron microscope–energy dispersive X-ray spectroscopy (SEM-EDX) instrument, FELMI-ZFE, Graz, Austria; prior to analysis, the samples were coated with gold to make them conductive, which prevents electrical charging and improves image quality.

## 3. Results and Discussion

### 3.1. Thermal Properties of the Hybrid Polymeric Films

TGA and DSC analyses were carried out to evaluate the thermal properties and thermal stability of the prepared hybrid polymeric films with increasing temperature. The differences in the thermal behavior of the samples were examined using the resulting thermograms. In the DSC analysis, endothermic peaks were observed in the range of 240–248 °C for all formulations, as shown in Figure 1. When the TGA curves in Figure 2 were examined, a clear mass loss was seen to begin in the same temperature range for all films, indicating that these endothermic peaks correspond to the onset of thermal degradation. The first-stage mass loss of 10–15% between 30 and 240 °C occurred due to the removal of water within the films and the decomposition of hydroxyl groups in the CMC [36]. The degradation process was found to continue up to about 300 °C, and the total mass loss in this range reached approximately 40–50%. This loss is attributed to the breakdown of the glycosidic bonds and carboxylate groups of the CMC [37]. While F1 (pure CMC) exhibited the highest endothermic peak temperature (248 °C), this value decreased slightly with the addition of nanocellulose (F2: 242 °C, F3: 240 °C). However, the formulations containing inorganic reinforcement (F4: 246 °C, F7: 247 °C) maintained peak temperatures close to that of the reference film. This endothermic transition corresponds to the onset of degradation of the crystalline regions of the CMC matrix and nanocellulose, as further confirmed by the overlapping first DTGA peak (Section 3.1, Figure 3). As this transition is an intrinsic feature of the CMC/nanocellulose crystalline domains and is not expected to shift substantially with filler content, the DSC results alone do not indicate whether thermal stability has improved; the evidence for a thermal barrier effect is provided by the TGA and DTGA results discussed below.

DTGA curves are obtained by taking the first derivative of the TGA curves and show the temperature at which the mass loss occurs most rapidly. When the DTGA curves in Figure 3 were examined, two separate degradation peaks were observed at approximately 240–250 °C and 270–290 °C for all formulations, which is consistent with previous academic studies [38,39]. The first peak overlaps with the endothermic peaks detected in the DSC analysis and corresponds to the degradation of the crystalline regions of the CMC matrix and nanocellulose. The first peak is associated with the decarboxylation and decomposition of the carboxyl groups (COO^−^) in the molecular structure of CMC [40]. Consistent with this two-stage degradation behavior, 50% of the total mass loss of the pure CMC film (F1) was completed at 303 °C (Table 2). The second peak reflects the thermal breakdown of the main CMC chain [40]. The lower DTGA peak intensities of the formulations containing inorganic reinforcement (F4, F5, F6, F7) compared to the reference film F1 indicate that bentonite and ground ceramic waste reduce the degradation rate and improve thermal stability.

In contrast to the DSC peak temperature, the TGA-derived parameters directly quantify the extent and rate of mass loss and therefore provide more reliable evidence of thermal stability. When Table 2 is examined, the pure CMC film (F1) showed the lowest T_10_% value (150 °C), which largely reflects the release of absorbed moisture together with early-stage degradation, whereas this value increased markedly in the formulations containing inorganic reinforcement. In particular, the T_10_% values of the F6 and F7 films containing ground ceramic waste reached 207 °C and 203 °C, respectively, while the highest T_50_% value was recorded for F7 at 397 °C. This distinction reflects the fact that T10% and T50% probe different stages of the degradation process. T10% is primarily governed by early-stage mass loss, where the lower ceramic waste loading in F6 (5 wt.%) is more homogeneously dispersed within the matrix (Section 3.4) and therefore delays the onset of degradation more effectively at low temperature. T50%, in contrast, reflects the main-chain degradation regime at higher temperature, where the larger inorganic fraction present in F7 (10 wt.%) provides greater protection and a higher non-volatile residue. F6 and F7 therefore represent optimal formulations in different degradation regimes rather than following a single monotonic trend with filler content. These findings clearly show that bentonite and ground ceramic waste act as a thermal barrier within the CMC matrix and that thermal stability improves as the amount of inorganic phase increases [39]. The increase in char yield values (F1: 32.0% → F7: 40.2%) is consistent with the contribution of the inorganic reinforcement to the residual mass [28]; however, since part of this increase reflects the intrinsic non-combustible fraction of the added filler itself, char yield alone cannot be taken as direct evidence of a thermal barrier mechanism. The primary evidence for the barrier/retardation effect is instead provided by the kinetic parameters discussed above—the marked increases in T10% and T50% and the reduced DTGA peak intensities of the reinforced formulations relative to F1 (Section 3.1, Figure 3)—which reflect a genuine reduction in degradation rate rather than a passive residue effect.”

### 3.2. Thermomechanical Properties of the Hybrid Polymeric Films

When the storage modulus (E′) curves shown in Figure 4 were examined, the E′ values of films F1–F5 decreased with increasing temperature. This behavior is consistent with the expected viscoelastic character of polymeric materials, in which chain mobility increases with rising temperature [41]. Among the formulations, the highest E′ value (~25 GPa) was obtained from the F4 film containing nanocellulose (NC) and a low amount of bentonite, indicating that F4 showed the greatest dynamic stiffness under the applied oscillatory load. It should be noted that this reflects dynamic mechanical behavior under small-strain oscillatory deformation and does not by itself establish overall mechanical performance, which would require complementary quasi-static tests (e.g., tensile, flexural, or puncture testing). In the F5 film, the increased bentonite ratio caused particle agglomeration and resulted in a lower storage modulus. In addition, the relatively low E′ values of the F6 and F7 films containing ceramic waste can be explained by the weak interaction between the ground ceramic waste and the CMC matrix, together with the agglomeration effect. The weak interfacial bond caused the particles to slide under stress, reducing the stiffness of the film [42]. Another notable behavior is the relatively small change in the E′ values of the F6 and F7 films with increasing temperature, in contrast to the more pronounced softening observed for F1–F5. This behavior is consistent with the presence of rigid inorganic clusters that may restrict matrix deformation at elevated temperature, potentially through the formation of a filler network within the CMC matrix [43]. However, as no rheological measurements, electrical/conductivity analysis, or quantitative microstructural characterization (e.g., filler-network imaging) were performed in this study, the existence of a true percolation network cannot be confirmed from the DMA data alone and is proposed as a hypothesis for future investigation.

The loss modulus (E″) is an indicator of viscous behavior, expressing the amount of energy dissipated as heat by polymeric materials during deformation (Figure 5). The peak observed at around 60–80 °C in the E″ curves corresponds to a transition in the CMC matrix related to moisture loss and chain mobility. The F4 film had the highest loss modulus values (1.08–1.35 GPa) over the entire temperature range. This is explained by the increased interfacial friction created by the bentonite layers between the polymer chains; layered clays such as bentonite, thanks to their high surface areas, restrict the movement of the polymer chains and cause mechanical energy to be converted into heat during deformation [44]. In contrast, the F6 and F7 films exhibited the lowest loss modulus values (0.10–0.19 GPa). Unlike the storage modulus, which showed relative thermal stability in F6 and F7 (Section 3.2, attributed to rigid filler clusters restricting deformation), the loss modulus reflects the efficiency of energy dissipation at the filler–matrix interface. The low loss modulus of F6 and F7 is therefore attributed to weak interfacial adhesion between the ground ceramic waste and the CMC matrix, which limits interfacial friction and stress transfer and consequently reduces the amount of mechanical energy converted into heat. The decrease in the loss modulus of F5 similarly results from excessive bentonite loading, which causes agglomeration that reduces the effective filler–matrix contact area and prevents sufficient interaction with the polymer chains [45].

Tan delta (loss factor) is the ratio of the loss modulus (E″) to the storage modulus (E′) and is an important viscoelastic parameter that directly indicates the energy damping capacity of a polymeric material. When the data in Figure 6 are examined, the absence of a clear peak shows that the CMC-based films undergo moisture loss and thermal degradation rather than a glass transition in the 35–200 °C temperature range, which is consistent with the DSC and TGA results. In the tan delta curves, the F6 formulation exhibited the highest damping values over the entire temperature range. This indicates that adding a small amount of ceramic waste was not sufficient to create a homogeneous distribution and an effective reinforcement–matrix interface within the CMC matrix. It has been reported in the literature that, at amounts below the optimum reinforcement concentration, inorganic reinforcement can increase the energy loss factor by disrupting matrix homogeneity rather than restricting chain mobility [45]. Considering that the tan delta peak value decreases in systems with strong reinforcement–matrix interfacial interactions compared to systems with weak interactions [46], the high tan delta value of F6 shows that the ceramic waste could not establish sufficient interfacial interaction with the CMC matrix at this concentration.

On the other hand, the local minimum observed after ~160 °C in the tan delta curve of the F6 formulation, followed by a renewed increase, is attributed to the activation of more than one relaxation mechanism in different temperature ranges. The shoulder or multiple-peak structure observed in the tan delta curve of polymer composites and blends is an indicator of limited compatibility between the components and of differing levels of segmental mobility; in fully miscible systems, a single tan delta peak is expected. Accordingly, the wavy behavior in F6 is thought to result from the heterogeneous distribution of the small amount of ceramic waste within the matrix; as a result, it has been reported that the heterogeneous distribution of the inorganic reinforcement leads to different regional stiffness and segmental mobility values within the matrix, causing more than one relaxation mode to appear [41]. In the F7 formulation, in which the amount of reinforcement was increased, both the tan delta values decreased and this fluctuation disappeared; this finding suggests that, at the higher loading, the ceramic waste particles are distributed more continuously within the matrix—consistent with the more stable storage modulus discussed in Section 3.2—resulting in a more uniform relaxation response [45]. As noted above, this filler-network interpretation remains a hypothesis, as direct structural or rheological confirmation of a percolation network was not obtained in this study. In contrast, the F1 and F4 films exhibited a monotonic and smooth increase in tan delta thanks to their single-phase or well-dispersed reinforcement structure; this finding is consistent with studies reporting that uniform relaxation behavior is expected in miscible systems [46].

### 3.3. FT-IR Spectroscopy of the Hybrid Polymeric Films

When the FT-IR spectra in Figure 7 were examined, the characteristic bands specific to CMC were preserved in all hybrid films. Accordingly, the presence of the O–H stretching band at 3600–3000 cm^−1^, the aliphatic C–H stretching band at 2900 cm^−1^, the asymmetric COO^−^ stretching band at 1590 cm^−1^, the symmetric COO^−^ stretching and C–H bending bands at 1415 cm^−1^, and the C–O–C stretching band of the glycosidic bond at 1000 cm^−1^ in the CMC films is consistent with the literature [47]. In the nanocellulose-containing formulations (F2–F7), a reduction in the intensity of the O–H (3600–3000 cm^−1^), C–H (2900 cm^−1^), and asymmetric COO^−^ (1590 cm^−1^) bands was observed relative to the reference film (F1). Similar spectral behavior has previously been reported for CMC/nanocellulose systems and has been associated with interactions between the hydroxyl groups of the polymer matrix and the reinforcing phase [40]. However, because the present study is based solely on qualitative FT-IR observations, the existence of hydrogen bonding cannot be confirmed quantitatively, and additional analyses, such as peak deconvolution and peak-area integration, would be required. In the literature, the change in O–H band intensity in CMC/nanocellulose composite films has been reported as an indicator of the hydrogen bonding interaction between the biopolymer and NC [20,40]. In the bentonite-containing formulations (F4, F5), the appearance of the Si–O asymmetric stretching band at ~1027–1040 cm^−1^ and the Al–Si–O bending band at ~520 cm^−1^ confirms that the bentonite phase was incorporated into the film structure [48,49]. Similarly, in the ceramic-waste-containing formulations (F6, F7), the observation of the Si–O–Si asymmetric stretching band at ~1000–1050 cm^−1^ demonstrates the presence of the ceramic waste additive [50]. The absence of new characteristic bands in all formulations indicates that the interaction between the components is based on hydrogen bonding and physical mixing mechanisms rather than covalent bond formation [47,51].

### 3.4. Morphological Properties of the Hybrid Polymeric Films

The morphology of the hybrid polymeric films was examined by SEM. Figure 8 shows the SEM images at ×20,000 magnification. F1, the pure CMC film, gave a homogeneous and continuous image, consistent with the images reported in the literature [52]. In the F2 and F3 films prepared with the addition of nanocellulose, a denser and more fibrous structure formed, which results from nanocellulose creating a three-dimensional network structure within the polymer matrix [53]. In the bentonite-containing formulations (F4 and F5), an increase in surface roughness was observed, together with the appearance of irregular regions that may be associated with local filler accumulation. Similarly, the ceramic-waste-containing formulations (F6 and F7) exhibited increasingly heterogeneous morphologies with increasing filler content. These observations are qualitatively consistent with the possibility of filler agglomeration at higher loading levels. However, because no quantitative image analysis was performed, the extent of agglomeration and its relationship to the thermomechanical properties cannot be established conclusively. In general, it was determined that additives at low ratios were distributed more homogeneously within the matrix, while at high ratios the surface irregularity increased due to agglomeration.

EDX analyses were carried out to determine the chemical composition on the surfaces of the fractured samples. The EDX data are given in Table 3 as weight percent (wt%) and atomic percent (at%). In all films, carbon (C), oxygen (O), and sodium (Na) elements originating from the structure of the CMC matrix were detected. With the addition of nanocellulose (F2, F3), a marked increase in the oxygen ratio was observed compared to F1, due to the hydroxyl groups in the structure of the nanocellulose. In the bentonite-containing films (F4, F5), silicon (Si) and aluminum (Al) elements originating from the silica (SiO_2_) and alumina (Al_2_O_3_) in the bentonite structure were detected; in particular, the Al content reached 17.53 wt% in F5. This value is considerably higher than expected from the bulk composition of bentonite, which is Si-rich (SiO_2_ ≫ Al_2_O_3_), and indicates that the analyzed region coincided with a local bentonite agglomerate, in agreement with the agglomeration tendency observed in the SEM images of F5; magnesium (Mg) originating from the bentonite structure was also detected. In the ceramic-waste-containing films (F6, F7), Si and Al elements were likewise detected, and with the increasing reinforcement ratio, an increase in the Si and Al ratios was observed in F7 compared to F6. Considering the localized nature of EDX point analysis, the detected elements were consistent with the compositions of the hybrid polymeric films.

## 4. Conclusions

In this study, hybrid polymeric films were prepared in seven different formulations by adding nanocellulose, bentonite, and ground ceramic waste reinforcements to a CMC matrix, and their properties were characterized. The films were produced by the casting method, and their thermal and thermomechanical behavior was examined by TGA/DTGA, DSC, and DMA; their morphology and particle distribution by SEM-EDX; and their chemical structure by FT-IR spectroscopy. The DSC endothermic peaks, evaluated together with the TGA results, corresponded to the onset of thermal degradation, while the two DTGA peaks observed for all formulations revealed the two-stage degradation behavior of CMC. The reduced peak intensities and the improved thermal stability with increasing inorganic content confirmed that bentonite and ground ceramic waste act as a thermal barrier within the CMC matrix, a conclusion further supported by the increase in char yield. The DMA results showed that the combination of nanocellulose and a low amount of bentonite maximized dynamic stiffness and energy dissipation capacity under oscillatory loading, whereas excessive bentonite loading reduced these properties through agglomeration; in the ceramic-waste-containing films, the formation of a percolation network kept the storage modulus relatively stable at high temperatures despite the weak matrix–reinforcement interface. The FT-IR spectra confirmed that the inorganic additives did not disrupt the chemical structure of CMC and suggested that the interactions between the components were mainly governed by physical interactions, while the possible contribution of hydrogen bonding requires further quantitative investigation. SEM-EDX observations suggested a more homogeneous distribution of the reinforcing phases at lower loading levels; however, quantitative image analysis would be required to confirm this interpretation. Overall, CMC-based hybrid polymeric films were successfully produced with the aim of developing a biodegradable and environmentally friendly alternative to petroleum-based plastics while also valorizing waste from the vitrified ceramic industry. Among the formulations, F4—containing nanocellulose and a low amount of bentonite—stood out in terms of dynamic thermomechanical performance with the highest storage and loss modulus values, whereas F7—containing a high amount of ceramic waste—exhibited the best thermal stability. Accordingly, F4 can be regarded as a promising formulation for applications requiring high dynamic stiffness, pending confirmation by quasi-static mechanical testing (e.g., tensile strength, elongation at break, and puncture/tear resistance), which is proposed as future work. Thanks to their biodegradable nature and the use of waste material, the developed films offer a promising alternative for sustainable applications such as food packaging, surface coating, and biomedical uses.

## Figures and Tables

**Figure 1:**
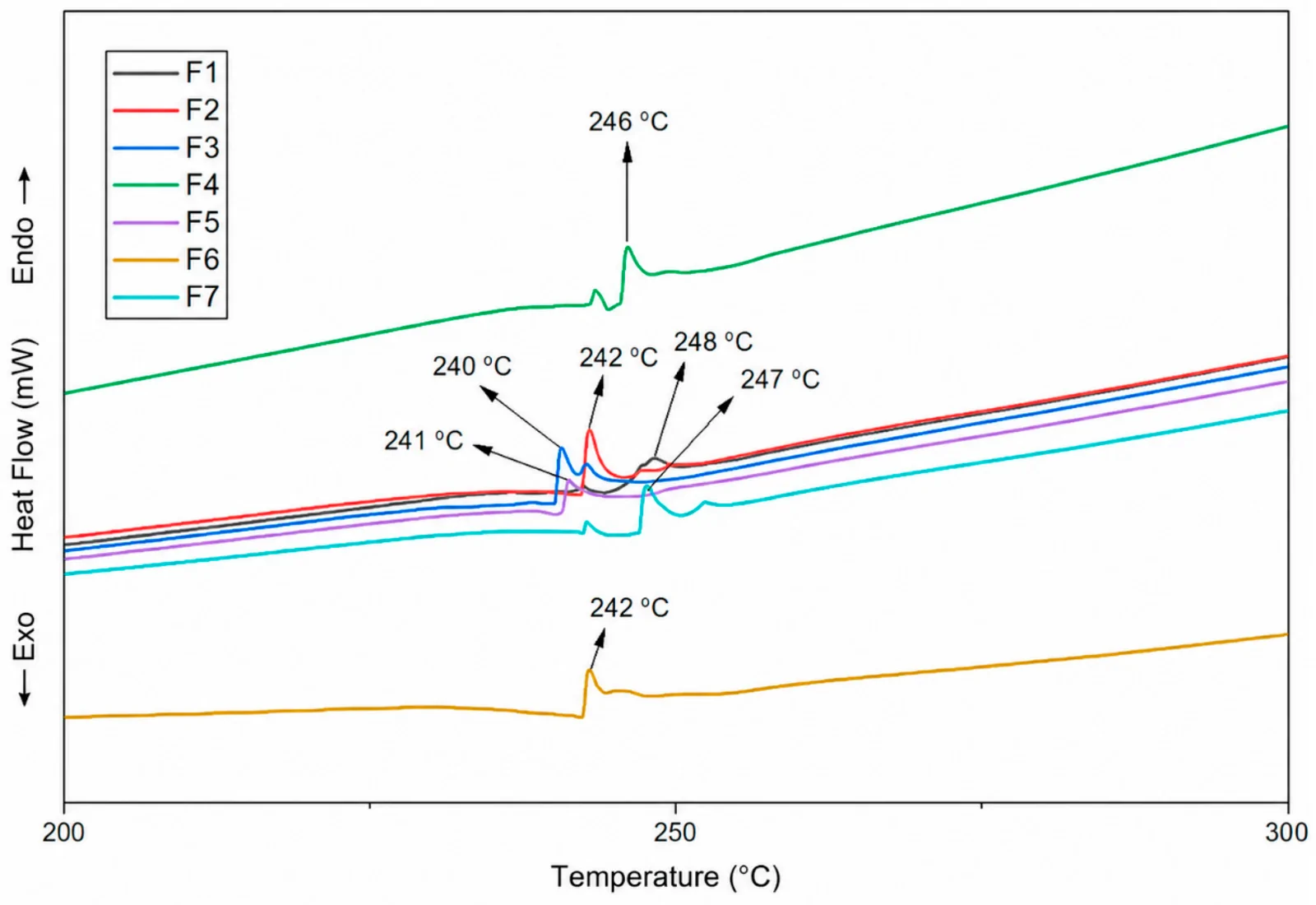
DSC thermograms of the hybrid polymeric films.

**Figure 2:**
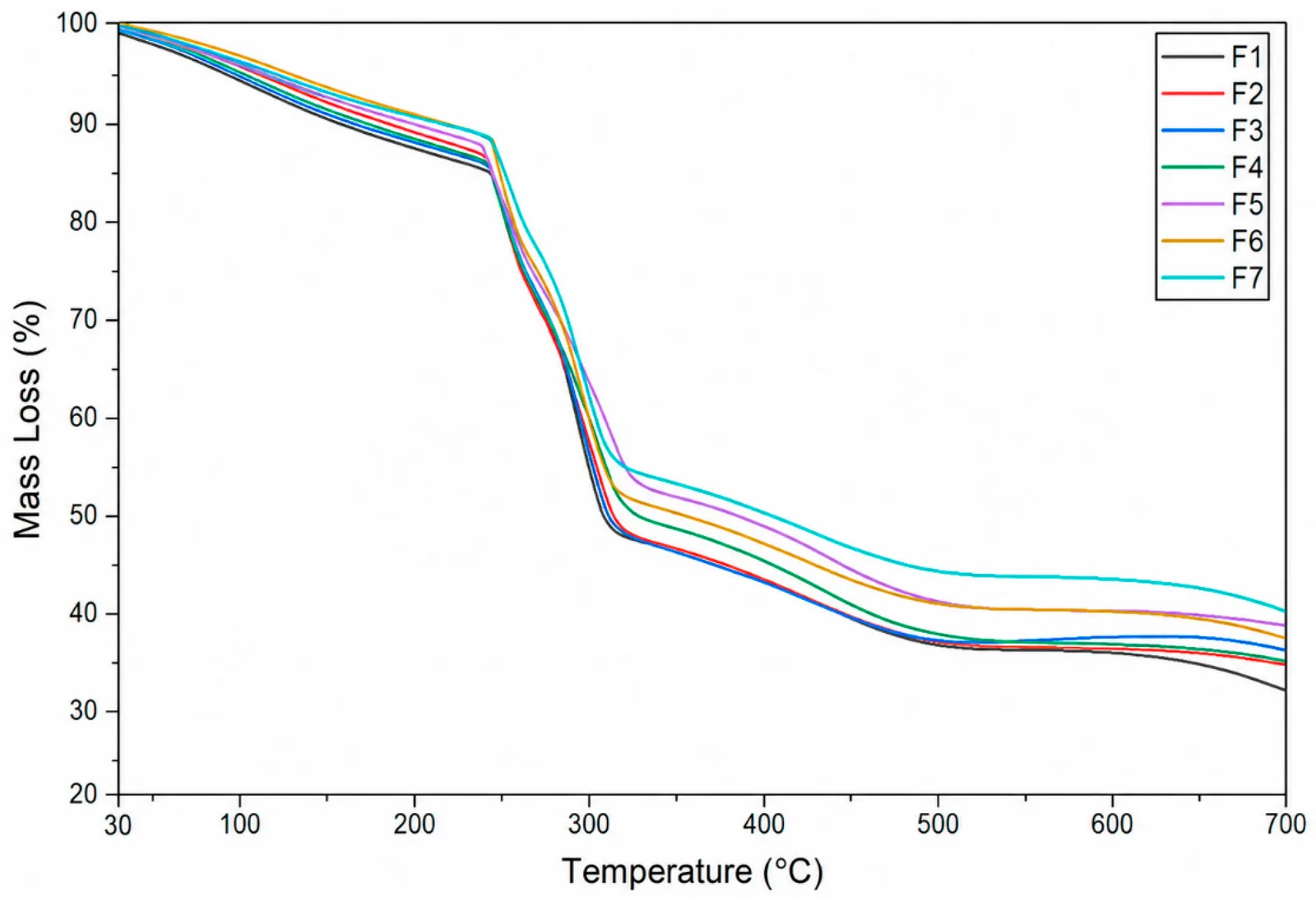
TGA curves of the hybrid polymeric films.

**Figure 3:**
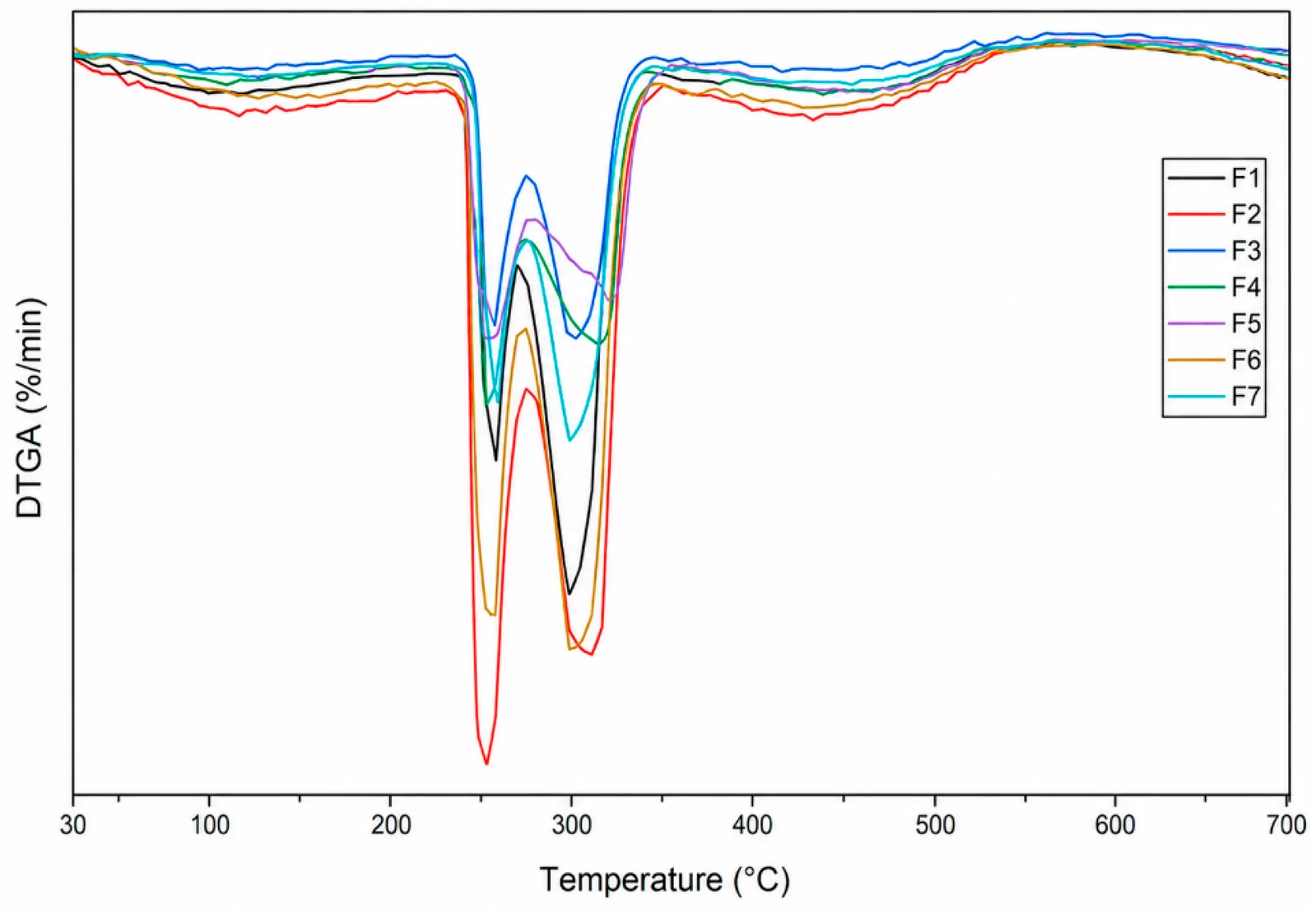
DTGA curves of the hybrid polymeric films.

**Figure 4:**
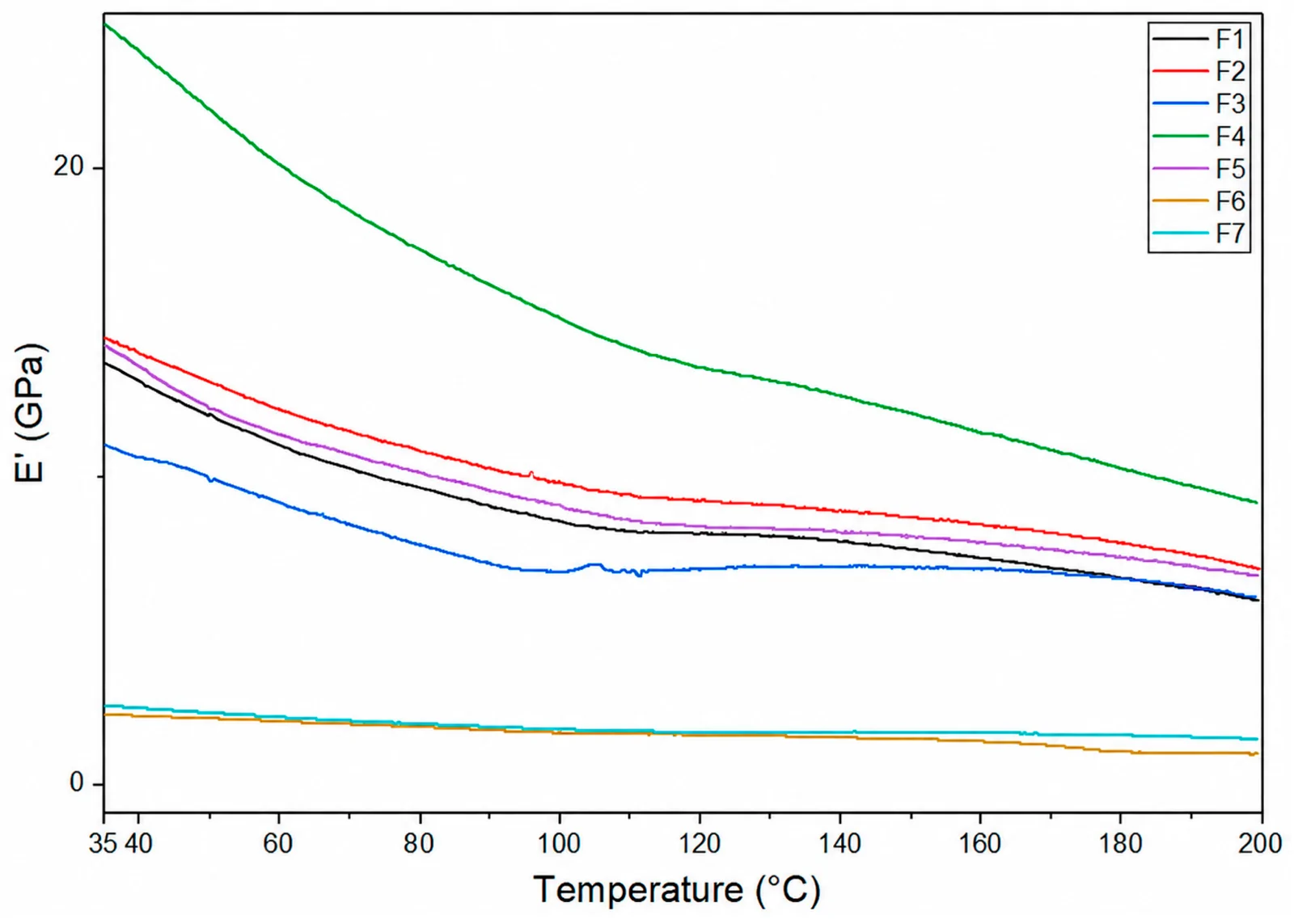
Storage modulus (E′) curves of the hybrid polymeric films.

**Figure 5:**
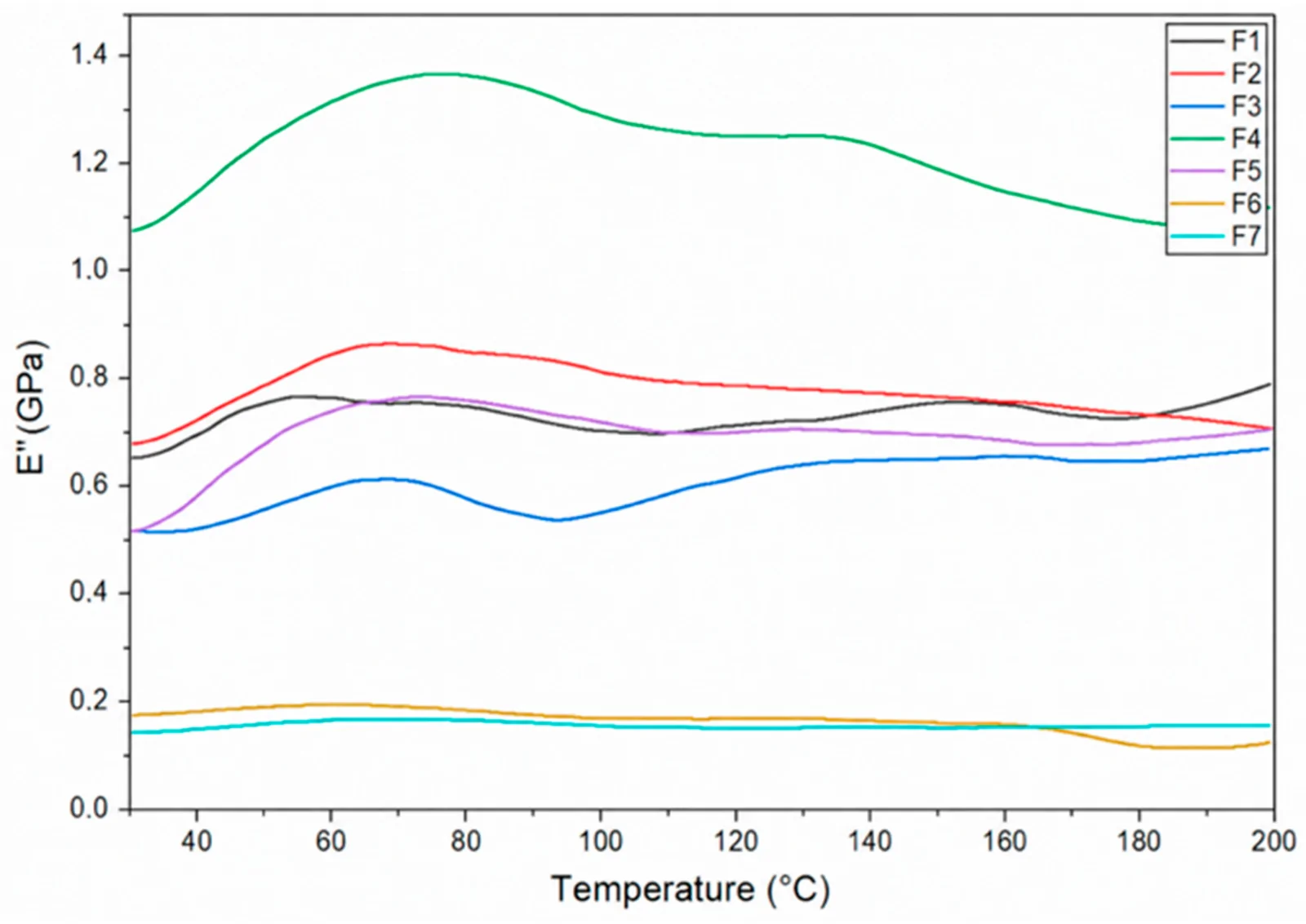
Loss modulus (E″) curves of the hybrid polymeric films.

**Figure 6:**
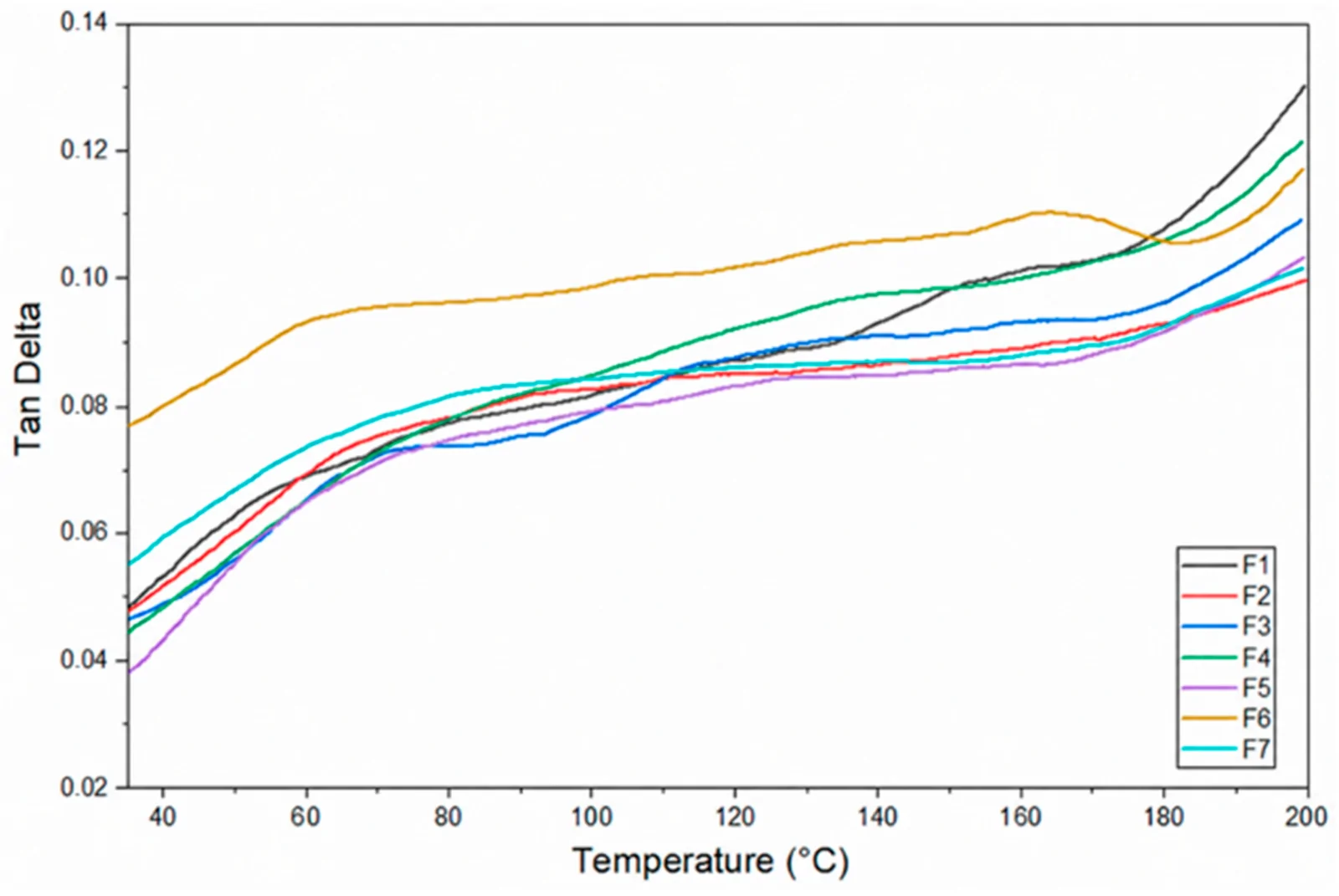
Tan delta curves of the hybrid polymeric films.

**Figure 7:**
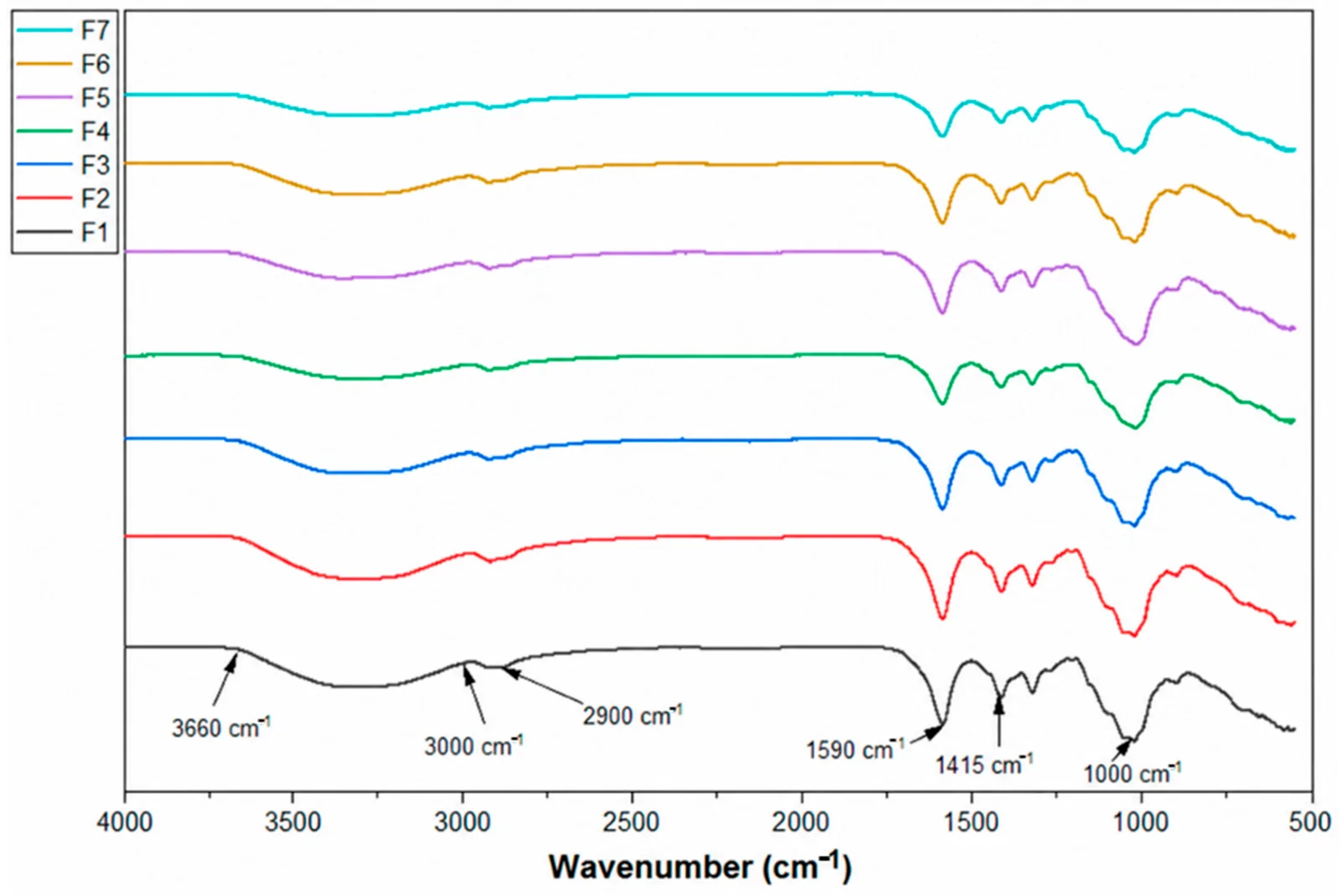
FT-IR spectra of the hybrid polymeric films.

**Figure 8:**
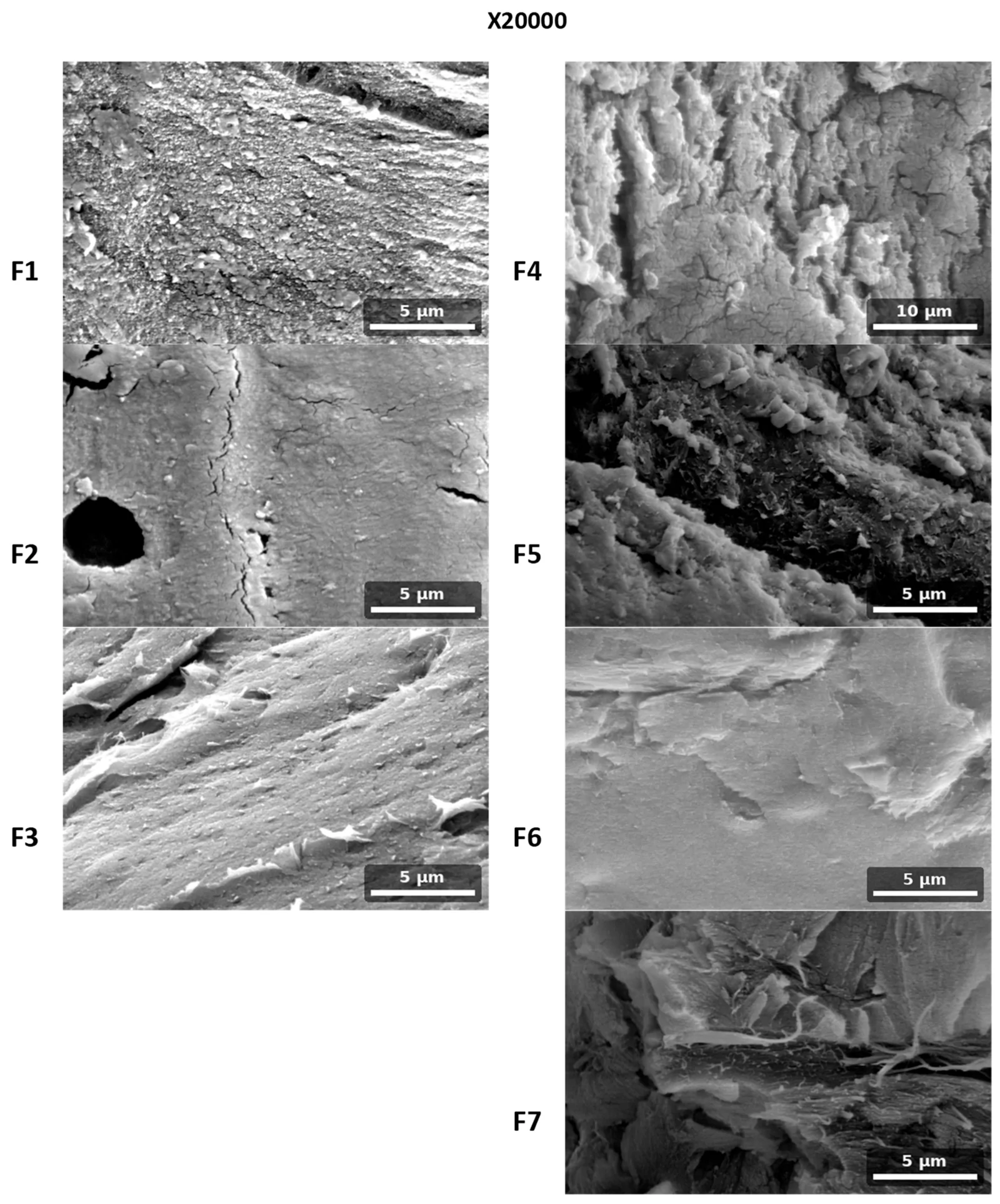
SEM images of the hybrid polymeric films at ×20,000 magnification.

**Table 1:** Formulation of hybrid polymeric films.

Sample	CMC (%)	NC (%)	Bentonite (%)	Ground Ceramic Waste (%)
F1	100	--	-	-
F2	95	5	-	-
F3	90	10	-	-
F4	85	10	5	-
F5	80	10	10	-
F6	85	10	-	5
F7	80	10	-	10

**Table 2:** Mass loss data of the hybrid polymeric films.

Sample	T_10%_ (°C)	T_50%_ (°C)	Max. Mass Loss (°C)	Char Yield (%)
F1	150	303	288	32.0
F2	176	309	245	34.6
F3	154	307	292	36.2
F4	165	319	244	35.0
F5	192	377	245	38.6
F6	207	346	288	37.4
F7	203	397	288	40.2

**Table 3:** EDX data of the hybrid polymeric films.

		F1		F2		F3		
	Element	Wt(%)	At(%)	Wt(%)	At(%)	Wt(%)	At(%)	
	C	39.29	55.31	36.01	49.14	38.11	48.49	
	O	34.49	36.45	41.66	42.68	47.54	45.41	
	Na	8.68	6.39	5.83	4.16	8.06	5.36	
	F4		F5		F6		F7	
Element	Wt(%)	At(%)	Wt(%)	At(%)	Wt(%)	At(%)	Wt(%)	At(%)
C	41.29	52.62	32.40	46.52	38.00	48.89	34.68	46.37
O	43.98	42.08	35.09	37.82	46.19	44.61	45.82	45.99
Na	6.12	4.08	2.70	2.02	6.58	4.42	6.46	4.52
Si	0.45	0.25	1.01	0.62	1.13	0.62	1.30	0.74
Mg			1.32	0.94				
Al			17.53	11.20	1.68	0.96	2.76	1.64

## Data Availability

The original contributions presented in this study are included in the article. Further inquiries can be directed to the corresponding authors.

## References

[1] Odian G. (2004). Principles of Polymerization.

[2] Young R.J., Lovell P.A. (2011). Introduction to Polymers.

[3] Andrady A.L., Neal M.A. (2009). Applications and societal benefits of plastics. Philos. Trans. R. Soc. B Biol. Sci..

[4] Leja K., Lewandowicz G. (2010). Polymer biodegradation and biodegradable polymers—A review. Pol. J. Environ. Stud..

[5] Dallaev R., Papež N., Allaham M.M., Holcman V. (2025). Biodegradable polymers: Properties, applications, and environmental impact. Polymers.

[6] Jambeck J.R., Geyer R., Wilcox C., Siegler T.R., Perryman M., Andrady A., Narayan R., Law K.L. (2015). Plastic waste inputs from land into the ocean. Science.

[7] Geyer R., Jambeck J.R., Law K.L. (2017). Production, use, and fate of all plastics ever made. Sci. Adv..

[8] Sander M., Kohler H.-P.E., McNeill K. (2024). Polymer biodegradability 2.0. Synthetic Biodegradable and Biobased Polymers.

[9] UNEP (2023). Turning off the Tap: How the World Can End Plastic Pollution and Create a Circular Economy.

[10] Siracusa V., Rocculi P., Romani S., Dalla Rosa M. (2008). Biodegradable polymers for food packaging: A review. Trends Food Sci. Technol..

[11] Niaounakis M. (2015). Biopolymers: Processing and Products.

[12] Rahman M.S., Hasan M.S., Nitai A.S., Nam S., Karmakar A.K., Ahsan M.S., Shiddiky M.J.A., Ahmed M.B. (2021). Recent developments of carboxymethyl cellulose. Polymers.

[13] Klemm D., Heublein B., Fink H.P., Bohn A. (2005). Cellulose: Fascinating biopolymer and sustainable raw material. Angew. Chem. Int. Ed..

[14] Heinze T., Koschella A. (2005). Carboxymethyl ethers of cellulose and starch—A review. Macromol. Symp..

[15] Moon R.J., Martini A., Nairn J., Simonsen J., Youngblood J. (2011). Cellulose nanomaterials review: Structure, properties and nanocomposites. Chem. Soc. Rev..

[16] Churam T., Usubharatana P., Phungrassami H. (2024). Sustainable production of carboxymethyl cellulose: A biopolymer alternative from sugarcane (*Saccharum officinarum* L.) Leaves. Sustainability.

[17] Akhlaq M., Mushtaq U., Naz S., Uroos M. (2023). Carboxymethyl cellulose-based materials as an alternative source for sustainable electrochemical devices: A review. RSC Adv..

[18] Zhang Y., Chen Y., Wang H., Zhang Y. (2022). Carboxymethyl cellulose-based hydrogels: Synthesis and applications. Gels.

[19] Sothornvit R., Krochta J.M., Han J.H. (2005). Plasticizers in edible films and coatings. Innovations in Food Packaging.

[20] Azeredo H.M., Rosa M.F., Mattoso L.H.C. (2017). Nanocellulose in bio-based food packaging applications. Ind. Crops Prod..

[21] Klemm D., Kramer F., Moritz S., Lindström T., Ankerfors M., Gray D., Dorris A. (2011). Nanocelluloses: A new family of nature-based materials. Angew. Chem. Int. Ed..

[22] Dufresne A. (2013). Nanocellulose: A new ageless bionanomaterial. Mater. Today.

[23] Trache D., Hussin M.H., Haafiz M.K.M., Thakur V.K. (2017). Recent progress in cellulose nanocrystals: Sources and production. Nanoscale.

[24] Bergaya F., Lagaly G. (2013). Handbook of Clay Science.

[25] Pavlidou S., Papaspyrides C.D. (2008). A review on polymer–layered silicate nanocomposites. Prog. Polym. Sci..

[26] Ray S.S., Okamoto M. (2003). Polymer/layered silicate nanocomposites: A review from preparation to processing. Prog. Polym. Sci..

[27] Benslimane A., Bahlouli I.M., Bekkour K., Hammiche D. (2016). Thermal gelation properties of carboxymethyl cellulose and bentonite-carboxymethyl cellulose dispersions: Rheological considerations. Appl. Clay Sci..

[28] Mapossa A.B., da Silva Júnior A.H., De Oliveira C.R.S., Mhike W. (2023). Thermal, morphological and mechanical properties of multifunctional composites based on biodegradable polymers/bentonite clay: A review. Polymers.

[29] Zhang P., Zhang P., Wu J., Guo Z., Zhang Y., Zheng Y. (2023). Mechanical properties and durability of sustainable concrete manufactured using ceramic waste: A review. J. Renew. Mater..

[30] Al-Fakih A., Odeh A., Mahamood M.A.A., Al-Shugaa M.A., Al-Osta M.A., Ahmad S. (2023). Review of the properties of sustainable cementitious systems incorporating ceramic waste. Buildings.

[31] Alotaibi J.G., Alajmi A.E., Alsaeed T., Khalaf J.A., Yousif B.F. (2024). On the incorporation of waste ceramic powder into concrete. Front. Mech. Eng..

[32] Sakthi Balan G., Prakash B., Aravind Raj S., Karthik S., Mohan Kumar V. (2022). Analysis on the impact of recycled ceramic waste, fiber format, and manufacturing techniques on polymer composite properties. Eng. Res. Express.

[33] Victor S.P., Muthu J. (2014). Polymer ceramic composite materials for orthopedic applications—Relevance and need for mechanical match and bone regeneration. J. Mechatron..

[34] Seydibeyoğlu M.Ö., Doğru A., Wang J., Rencheck M., Han Y., Wang L., Gardner D.J. (2023). Review on hybrid reinforced polymer matrix composites with nanocellulose, nanomaterials, and other fibers. Polymers.

[35] Candan Z., Gardner D.J., Shaler S.M. (2016). Dynamic mechanical thermal analysis (DMTA) of cellulose nanofibril/nanoclay/pMDI nanocomposites. Compos. Part B Eng..

[36] Saadiah M.A., Zhang D., Nagao Y., Muzakir S.K., Samsudin A.S. (2019). Reducing crystallinity on thin film based CMC/PVA hybrid polymer for application as a host in polymer electrolytes. J. Non-Cryst. Solids.

[37] El-Sayed S., Mahmoud K.H., Fatah A.A., Hassen A. (2011). DSC, TGA and dielectric properties of carboxymethyl cellulose/polyvinyl alcohol blends. Phys. B Condens. Matter.

[38] El-Sakhawy M., Tohamy H.A.S., Salama A., Kamel S. (2019). Thermal properties of carboxymethyl cellulose acetate butyrate. Cellul. Chem. Technol..

[39] Suppiah K., Leng T.P., Husseinsyah S., Rahman R., Keat Y.C., Heng C.W. (2019). Thermal properties of carboxymethyl cellulose (CMC) filled halloysite nanotube (HNT) bio-nanocomposite films. Mater. Today Proc..

[40] Mandal A., Chakrabarty D. (2019). Studies on mechanical, thermal, and barrier properties of carboxymethyl cellulose film highly filled with nanocellulose. J. Thermoplast. Compos. Mater..

[41] Das Gupta A. (2025). DMA: Dynamic mechanical analysis. Advances in Polymer Science.

[42] Brostow W., Datashvili T., Geodakyan J., Lou J. (2011). Thermal and mechanical properties of EPDM/PP + thermal shock-resistant ceramic composites. J. Mater. Sci..

[43] Handge U.A., Wolff M.F., Abetz V., Heinrich S. (2016). Viscoelastic and dielectric properties of composites of poly(vinyl butyral) and alumina particles with a high filling degree. Polymer.

[44] Galimberti M., Kumar V., Coombs M., Cipolletti V., Agnelli S., Pandini S., Conzatti L. (2014). Filler networking of a nanographite with a high shape anisotropy and synergism with carbon black in poly(1,4-cis-isoprene)–based nanocomposites. Rubber Chem. Technol..

[45] Deng H., Fu Q. (2017). Recent progress on the confinement, assembly, and relaxation of inorganic functional fillers in polymer matrix during processing. Macromol. Rapid Commun..

[46] Bashir M.A. (2021). Use of dynamic mechanical analysis (DMA) for characterizing interfacial interactions in filled polymers. Solids.

[47] Badry R., El-Nahass M.M., Nada N., Elhaes H., Ibrahim M.A. (2023). Structural and UV-blocking properties of carboxymethyl cellulose sodium/CuO nanocomposite films. Sci. Rep..

[48] Paluszkiewicz C., Holtzer M., Bobrowski A. (2008). FTIR analysis of bentonite in moulding sands. J. Mol. Struct..

[49] Ravindra Reddy T., Kaneko S., Endo T., Lakshmi Reddy S. (2017). Spectroscopic characterization of bentonite. J. Lasers Opt. Photonics.

[50] Cherrat A., Mastouri H., El Kanzaoui M., Derraz M., Erradi E.M., Labjar N., Ennawaoui C. (2026). Performance optimization of ceramic-waste-based composite materials for structural applications. J. Compos. Sci..

[51] Hoque M., Hossain M.S., Hasan M.N., Jubayer M., Aktaruzzaman M. (2023). Investigating the characteristics of carboxymethyl cellulose film as a possible material for green packaging. GSC Biol. Pharm. Sci..

[52] He Y., Fei X., Li H. (2020). Carboxymethyl cellulose-based nanocomposites reinforced with montmorillonite and ε-poly-L-lysine for antimicrobial active food packaging. J. Appl. Polym. Sci..

[53] Jankowska I., Bielejewski M., Ławniczak P., Pankiewicz R., Brus J., Tritt-Goc J. (2025). Cellulose nanofibers vs. cellulose nanocrystals: A comparative study on their influence on the properties of imidazole-doped proton-conducting nanocomposites. Cellulose.

